# Low-temperature corn straw-degrading bacterial agent and moisture effects on indigenous microbes

**DOI:** 10.1007/s00253-023-12644-8

**Published:** 2023-07-01

**Authors:** Sainan Zhang, Shengcai Han, Julin Gao, Xiaofang Yu, Shuping Hu

**Affiliations:** 1 College of Agriculture, Inner Mongolia Agricultural University, 306 Zhaowunda Road, Saihan District, Inner Mongolia Autonomous Region 010000 Hohhot, People’s Republic of China; 2 Key Laboratory of Crop Cultivation and Genetic Improvement, Inner Mongolia Autonomous Region 010000 Hohhot, People’s Republic of China; 3 College of Horticulture and Plant Protection, Inner Mongolia Agricultural University, Hohhot, 010000 People’s Republic of China

**Keywords:** Corn straw, Low-temperature degradation, Soil moisture, Indigenous microorganism, Microbial network

## Abstract

**Abstract:**

While the in situ return of corn straw can improve soil fertility and farmland ecology, additional bacterial agents are required in low-temperature areas of northern China to accelerate straw degradation. Moisture is an important factor affecting microbial activity; however, owing to a lack of bacterial agents adapted to low-temperature complex soil environments, the effects of soil moisture on the interaction between exogenous bacterial agents and indigenous soil microorganisms remain unclear. To this end, we explored the effect of the compound bacterial agent CFF constructed using *Pseudomonas putida* and *Acinetobacter lwoffii*, developed to degrade corn straw in low-temperature soils (15 °C), on indigenous bacterial and fungal communities under dry (10% moisture content), slightly wet (20%), and wet (30%) soil-moisture conditions. The results showed that CFF application significantly affected the α-diversity of bacterial communities and changed both bacterial and fungal community structures, enhancing the correlation between microbial communities and soil-moisture content. CFF application also changed the network structure and the species of key microbial taxa, promoting more linkages among microbial genera. Notably, with an increase in soil moisture, CFF enhanced the rate of corn straw degradation by inducing positive interactions between bacterial and fungal genera and enriching straw degradation-related microbial taxa. Overall, our study demonstrates the alteration of indigenous microbial communities using bacterial agents (CFF) to overcome the limitations of indigenous microorganisms for in situ straw-return agriculture in low-temperature areas.

**Key points:**

*• Low-temperature and variable moisture conditions (10–30%) were compared*

*• Soil microbial network structure and linkages between genera were altered*

*• CFF improves straw degradation via positive interactions between soil microbes*

**Supplementary Information:**

The online version contains supplementary material available at 10.1007/s00253-023-12644-8.

## Introduction

With the ongoing development of global agriculture, discarded crop straw severely impacts agricultural sustainability and the wider environment (Harrison et al. 2020; Chen et al. 2021). In situ straw return is an effective way to recycle straw resources, having the advantages of increasing soil fertility and regulating the soil microbial environment (Yang et al. 2020). However, straw is not easily degraded under natural conditions, especially in low-temperature areas, implying that the application of in situ processing technology can be severely limited (Gong et al. 2020; Zheng et al. 2020). Consequently, exogenous high-efficiency bacterial agents that can efficiently degrade corn straw under low-temperature conditions have gained significant interest (Arntzen et al. 2020).

For instance, Chu et al. (2021) used *Phanerochaete* and *Chrysosporium* species to construct combined bacteria and reported corn straw lignin, cellulose, and hemicellulose degradation rates of 43.36%, 31.29%, and 48.36%, respectively, under optimum conditions (32 °C) after 20 days. The same authors revealed that coordination among microbial communities was key during corn straw degradation. Puentes-Téllez and Falcao Salles (2018) developed minimal active microbial consortia (MAMC; containing 18 lignocellulose-degrading strains) that degraded corn stover up to 96.5% at 28 °C, aided by the functional diversity and metabolic complementarity of MAMC. Compared with single strains, microbial consortia is well recognized as more adaptable, have better interactions, and achieve more effective degradation; however, the most favorable degradation results have been obtained at medium or high temperatures, and the research has focused on the straw degradation mechanism of added microbial consortia (AMC) in the absence of external microorganisms (Ding et al. 2019; Vassilev et al. 2020). Crucially, in practical applications, the degradation effects of AMC are largely influenced by indigenous microorganisms (Rillig and Mansour 2017; Wu et al. 2020), and the stability of indigenous soil microorganisms implies that degradation efficacy is largely limited by environmental conditions (Ratzke et al. 2020). Therefore, examining how AMC might alter indigenous microbial communities is important, particularly community structures linked to straw degradation, to optimize straw-return agriculture.

Soil bacteria and fungi are involved in a variety of critical ecological processes, and their diversity and community structure influence key soil characteristics, such as nutrient content, function, and stability (Koner et al. 2022). Moisture is an important factor affecting the structure and activity of soil microorganisms (Preece et al. 2019; Ouyang and Li 2020). Bacteria and fungi certainly form complex ecological networks in soil environments with different moisture contents, forming competitive or mutually beneficial associations between and within species. Furthermore, key communities are reportedly strongly associated with soil function (Tan et al. 2021). Nevertheless, the lack of suitable bacterial agents implies that relatively a few studies have been dedicated to their effects on indigenous soil microorganisms in low-temperature environments, specifically under different soil-moisture conditions.

In order to accelerate the degradation of corn straw, we screened two strains in the early stage of the experiment GF-40 and GF-45, which have the ability to degrade corn stover at low temperatures, and were identified as *Pseudomonas putida* and *Acinetobacter lwoffii*, respectively. They also have the advantages of lignocellulose production, low nitrogen tolerance, and wide pH adaptability. Therefore, we prepared these two bacteria with additives as a powdered solid bacterial agent CFF using freeze-drying technology in order for the strain to be better adapted to complex soil environments. Accordingly, in this study, we hypothesized that the exogenous application of bacterial agent CFF degrades corn straw by altering the community structure of indigenous microorganisms, enriching the indigenous microorganisms with straw degradation ability. This capacity of CFF to alter the microorganism community structure may be closely associated with soil moisture. Therefore, we investigated the degradation effect of compound bacterial agent CFF at different soil-moisture levels under low-temperature conditions (15 °C); we also examined changes in the diversity, structure, as well as intra- and inter-species interactions of indigenous microbial communities following CFF application. Furthermore, we compared these effects under different soil-moisture conditions, aiming to reveal the mechanism underlying the interactions between exogenous bacterial agents and indigenous microorganisms in straw degradation under low-temperature conditions. Hence, we provide an experimental framework that might help to develop and apply bacterial agents to straw-return agriculture in low-temperature environments.

## Materials and methods

### CFF

CFF, the bacterial agent used in this study, comprises two equally proportioned strains: *Pseudomonas putida* and *Acinetobacter lwoffii*. Reference samples are deposited in the China General Microbiological Culture Collection Center (CGMCC, Nos. 20521 and 20,522, respectively). The GenBank accession numbers for nucleotide sequences of GF-40 and GF-45 are SUB11224186 GF-40 ON063331 and SUB11224468 GF-45 ON064992, respectively.

The source of GF-40 and GF-45 was a soil with 8 years of continuous straw return, located in Chilechuan Modern Agriculture Expo Park, Tumed Right Banner, Baotou City, Inner Mongolia, China. Screening of GF-40 and GF-45 by lignin or cellulose using the single carbon source approach was initiated using the combined dilution and restriction selection method.

We experimentally verified the straw degradation ability, enzyme activity, and suitable survival conditions of the two strains in the early stage of the experiment. Briefly, single bacterial inoculum was mixed with soil supernatant at a ratio of 1: 400 (v: v), and 1 g of corn straw was added. Both the GF-40 and GF-45 strains degraded 18.35% and 21.31% of straw, respectively, after 14 d at 15 °C, which was significantly higher than that of the control treatments (no bacterial solution applied). Both GF-40 and GF-45 had high laccase activity of 47.4 U/L and 101.1 U/L, respectively, and GF-45 also had the ability to produce cellulose and hemicellulase. In addition, GF-40 and GF-45 had strong low nitrogen tolerance, especially GF-45, which also had high reproductive efficiency under all three pH conditions (pH = 5, 7, and 9), indicating broad pH adaptability.

The precipitate (bacterial biomass) obtained after centrifugation of the fermentation broth was mixed with an additive made from corn straw powder, starch, and bran (ratio = 4:3:9) at a mass ratio of 1:4, i.e., 4 g of additive mixed with 1 g of precipitate, corn straw powder, starch, and bran being 1 g, 0.75 g, and 2.25 g, respectively, and powdered CFF was prepared by freeze-drying. The prepared CFF was stored at 4 ℃ after vacuum-packing and had a viable bacteria concentration of 7.0 × 10^10^ colony forming units per gram of powder.

### Corn straw

Corn straw was collected as mature straw after autumn harvest from the experimental field at the Corn Research Center, Inner Mongolia Agricultural University, and was cut into 3–5 cm pieces after washing and drying.

### Experimental design and sampling

Forty-five culture boxes (size: 10 × 10 × 12 cm, length × width × height) were filled with 800 g of soil each (soil depth = 10 cm). Eight grams of corn straw was placed at a depth of 5 cm in each culture box. The straw was packed in gauze bags with 5-mm-diameter holes for easier collection. The culture boxes were randomly assigned to three initial soil-moisture conditions: 10% (W1, dry), 20% (W2, slightly wet), and 30% (W3, wet; *n* = 15 for each group). Samples were not watered during the experiment and thus simulating one irrigation cycle. We sought to reflect field conditions as closely as possible, and the treatments purposefully did not represent unrealistic “extreme” conditions. The 15 culture boxes assigned to each soil condition were further randomly divided into a control (J0, no CFF) and four treatment groups (J1–J4) with the following straw (kg) to CFF (kg) application ratios: 800:0.5 (J1), 800:1 (J2), 800:2 (J3), and 800:3 (J4; equivalent to CFF application rates of approximately 0.5, 1, 2, and 3 kg/667 m^2^, respectively). According to this ratio, the mass of CFF applied in this laboratory soil culture test were 0.005 g, 0.01 g, 0.02 g, and 0.03 g for treatments J1, J2, J3, and J4, respectively. We use a 10,000 ppm balance to weigh the required bacterial agent, place the weighed bacterial agent in a tinfoil packet, and gently pinch and seal the tinfoil packet. CFF was evenly spread on the corn straw, which was then covered with soil. Subsequently, all of the culture boxes were placed in a low-temperature incubator at 15 °C to simulate a low-temperature environment. The experimental design is detailed in Table 1.

**Table 1:** Experimental design for corn straw degradation (straw weight: 8 g)

Soil moisture	Group		CFF application rate	Sample code	Number of samples	Applied amount (g)	Straw: CFF
10%	Control	C1	J0	C1	3	——	——
	Treatment	W1	J1	1	3	0.005	800:0.5
			J2	2	3	0.01	800:1
			J3	3	3	0.02	800:2
			J4	4	3	0.03	800:3
20%	Control	C2	J0	C2	3	——	——
	Treatment	W2	J1	5	3	0.005	800:0.5
			J2	6	3	0.01	800:1
			J3	7	3	0.02	800:2
			J4	8	3	0.03	800:3
30%	Control	C3	J0	C3	3	——	——
	Treatment	W3	J1	9	3	0.005	800:0.5
			J2	10	3	0.01	800:1
			J3	11	3	0.02	800:2
			J4	12	3	0.03	800:3

After 40 days, approximately 50 g of soil was collected within a 5-cm radius around the straw pack in each culture box, which was then mixed evenly for 16S and internal transcribed spacer (ITS) microbial sequencing. Each treated straw was placed into a sieve (60 mesh) and rinsed with running water to ensure that the straw will not be lost, and dried to determine the degradation rate, as follows (Serrano-Gamboa et al. 2019):

1 $$\mathrm{Straw degradation rate }\left(\mathrm{\%}\right)=\left({W}_{0}-{W}_{1}\right)/{W}_{0}\times 100\mathrm{\%}$$

where *W*_*0*_ is the mass of corn straw before degradation (g) and *W*_*1*_ is the mass of residual straw after degradation (g).

### Microbial diversity sequencing

The genomic DNA of the microbial communities was extracted from 45 soil samples using the E.Z.N.A.® soil DNA kit (Omega Bio-tek, Norcross, GA, USA), according to the instructions of the manufacturer. The DNA extract was checked by agarose gel electrophoresis (1%), and DNA concentration and purity were determined using a NanoDrop 2000 UV–vis spectrophotometer (Thermo Scientific, Wilmington, NC, USA). The hypervariable region V3–V4 of the bacterial 16S rRNA gene was amplified with the primer pair 338F (5ʹ-ACTCCTACGGGAGGCAGCAG-3ʹ) and 806R (5ʹ-GGACTACHVGGGTWTCTAAT-3ʹ); the hypervariable region ITS1 of the fungi ITS rRNA gene was amplified with the primers ITS1 (5ʹ-CTTGGTCATTTAGAGGAAGTAA-3ʹ) and ITS2R (5ʹ-GCTGCGTTCTTCATCGATGC-3ʹ). Paired-end sequencing was performed on an Illumina MiSeq PE300 platform (Illumina, San Diego, CA, USA) according to the standard protocols of Majorbio Bio-Pharm Technology Co., Ltd. (Shanghai, China).

The raw 16S rRNA and ITS rRNA gene sequencing reads were demultiplexed, quality-filtered by fastp version 0.20.0 (https://github.com/OpenGene/fastp) (Chen et al. 2018), and merged by FLASH version 1.2.7 (http://www.cbcb.umd.edu/software/flash) (Magoč et al. 201) with the following criteria: (i) the 300 bp reads were truncated at any site receiving an average quality score of < 20 over a 50 bp sliding window, and the truncated reads shorter than 50 bp were discarded; reads containing ambiguous characters were also discarded; (ii) only overlapping sequences longer than 10 bp were assembled according to their overlapped sequence. The maximum mismatch ratio of overlap region is 0.2. Reads that could not be assembled were discarded; (iii) samples were distinguished according to the barcode and primers, and the sequence direction was adjusted, exact barcode matching, 2 nucleotide mismatches in primer matching.

Operational taxonomic units (OTUs) with 97% similarity cutoff (Edgar 2013; Stackebrandt and Goebel 2014) were clustered using UPARSE version 7.1 (http://drive5.com/uparse/) (Edgar 2013), and chimeric sequences were identified and removed. The taxonomy of each OTU representative sequence was analyzed by RDP Classifier version 2.2 (http://rdp.cme.msu.edu/) (Wang et al. 2007) against the 16S rRNA database (e.g., Silva v138) and ITS rRNA database (e.g., Unite v8.0) using confidence threshold of 0.7.

All raw sequencing data (.fg files) were deposited in the NCBI Sequence Read Archive database under accession number PRJNA870440.

### Network analysis

SPSS 22.0 (IBM Corp., Armonk, NY, USA) was used to perform correlation analysis on the abundant (> 1%) bacterial and fungal genera. To reduce network complexity and improve computational accuracy (Soffer et al. 2014), bacterial or fungal genera with extremely significant (*P* < 0.01), strong correlations (Spearman’s correlation, *r* > 0.7) with other bacterial or fungal genera were selected (Xia et al. 2020). Subsequently, Cytoscape 3.7.1 (Shannon et al. 2003) was used to construct bacterial and fungal networks for the control and CFF-treated soils under the three moisture conditions (10%, 20%, and 30% moisture content). The “CytoNCA” tool was used to analyze and rank the degree (the connectivity indicates the number of nodes directly connected to a given node; higher connectivity means the node is more important in the overall network) and betweenness centrality (the role of a node in connecting to other nodes; the higher the value, the more important the role of the node in maintaining tight connectivity of the entire network) of the network nodes. The five genera with the highest betweenness centrality score (Vick-Majors et al. 2013) and a high degree of centrality (> 5) were considered key to network stability.

### Statistical analysis

All data were sorted using Excel 2010 (Microsoft, Redmond, WA, USA). Alpha diversity of the bacteria and fungi was tested for homogeneity of variance using Levene’s test, and α-diversity analysis was carried out using mothur (v.1.30.2, https://mothur.org/wiki/calculators/) at a 97% similarity at the operational taxonomic unit level. Analysis of variance (ANOVA) was performed using SPSS 22.0, and multiple comparisons between treatments were made using Tukey’s HSD test. Diversity boxplots were drawn using SigmaPlot 12.5 (Systat Software GmbH, Erkrath, Germany).

Unconstrained principal coordinates analysis (PCoA) based on the unweighted UniFrac distance matrix was performed using the citation (“vegan”) in R (version 3.3.1, R Foundation for Statistical Computing, Vienna, Austria) to visualize the bacterial and fungal community structures. Using the “Adonis” function in the “vegan” package, a permutational multivariate ANOVA (PERMANOVA) based on the Bray–Curtis dissimilarity matrix was also conducted.

Histograms of relative abundances of bacterial and fungal taxa at the phylum and genus levels across all treatments were plotted using SigmaPlot. The relationships between soil moisture and relative abundances of bacterial and fungal taxa at the phylum and genus levels were examined using Spearman’s correlation analysis in SPSS 22.0. When testing the differences between soil bacteria and fungi with or without CFF and under different soil-moisture conditions, the data were first tested for normality; a* t* test was applied to normally distributed data; otherwise, the non-parametric rank sum test was used.

ChiPlot (https://www.chiplot.online/) was used to analyze and plot the correlation between corn straw degradation rates and abundant bacterial and fungal genera under different treatment conditions.

## Results

### Analysis of corn straw degradation rate and soil microbial diversity

**Fig. 1:**
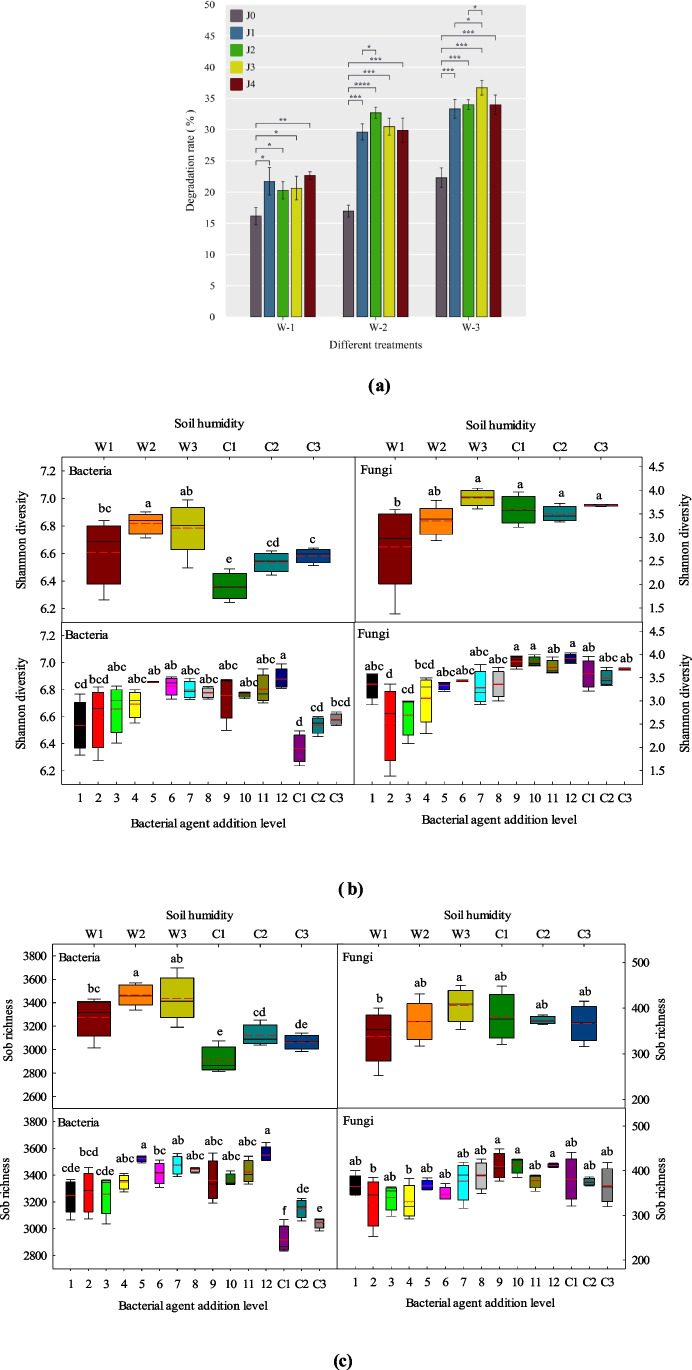

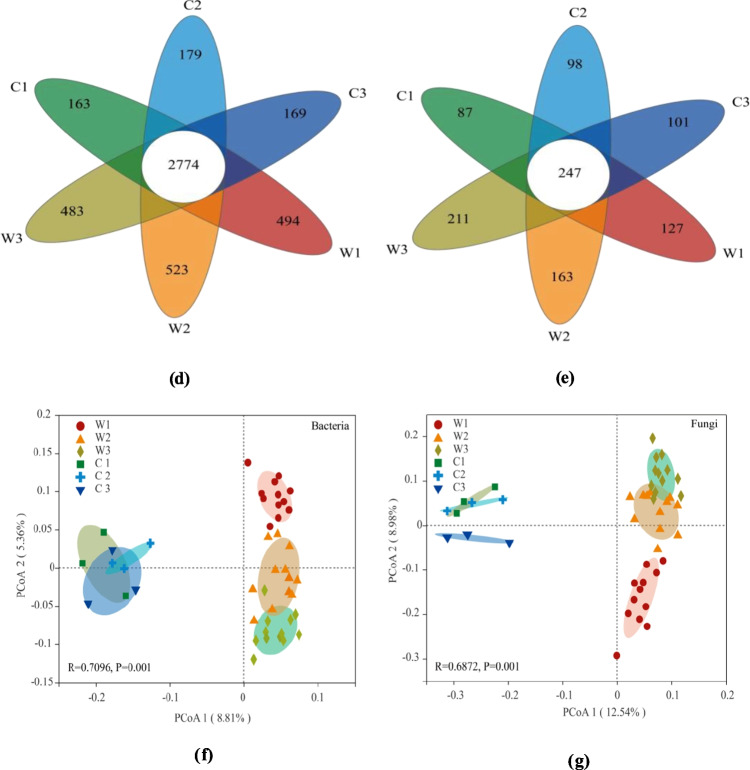
Degradation rates of corn straw and changes in indigenous bacterial and fungal α-diversity. **a** Degradation rates of corn straw; *P < 0.05, **P < 0.01, ***P < 0.001. **b** Shannon and **c** Sob indices of soil bacteria and fungi. All data are presented as mean (± SD), and different letter labels represent significant differences between treatments (*P* < 0.05). The number of species common and unique to different treatments of **d** soil bacteria **e** and fungi (OTU levels). Venn diagram shows the unique and shared OTUS (3% distance level) in different treatments. Different colors represent different treatments, and numbers in overlapping sections represent the number of shared species, while numbers in non-overlapping sections represent the number of unique species. **f** Principal coordinate analysis (PCoA) of bacterial and **g** fungal communities

The straw degradation rate improved with the increase in soil moisture, and under all soil-moisture conditions, the degradation rate was significantly higher after CFF application than in the control group (Fig. 1a). No marked differences in straw degradation rates were observed between CFF treatments of different ratios.

Figure 1b and c show the α-diversity of the soil microbial communities under different soil-moisture conditions and CFF application rates. In the analysis of all soil samples (under W1-3 and C1-3 treatments), the Shannon and Sob indices for bacteria were significantly higher (*P* < 0.05) for CFF-treated soils than for the control group under each moisture condition. Furthermore, these indices were higher for the slightly wet and wet soil (20% and 30%) conditions than for the dry soil (10%) treatment. Under the same soil-moisture conditions, the application of CFF (at any rate) significantly increased (*P* < 0.05) the bacterial Sob indices relative to the control group. Based on the Shannon index, the J3 and J4 treatments at 10% moisture content, J1 and J2 treatments at 20% moisture, and J4 treatment at 30% soil moisture yielded significant increases in bacterial diversity compared with the control group. In contrast, we found no significant differences (*P* > 0.05) in fungal richness or diversity between any of the treatments nor in comparison to the control. Under the same soil-moisture conditions, no significant difference in soil diversity was found between treatments with different CFF application rates.

Venn diagrams can be used to count the number of species shared and unique between samples, and it can reflect the similarity and specificity of species composition in the samples more visually. In this study, the OTUS level of 97% similarity was selected for Venn diagram analysis (Fig. 1d and e). The figure shows that the number of shared OTUs of soil bacteria and fungi under different treatment conditions were 2774 and 247, respectively. The unique numbers of soil bacteria and fungi in different soil-moisture conditions with the application of bacterial agent CFF treatment (W1-3) were higher than those in the control treatment (C1-3). Furthermore, a small difference was observed in the unique number of soil bacteria between soil-moisture conditions within the CFF treatment. Conversely, the difference is large among fungi, and the number of unique fungi OTUs increased gradually with the increase in soil moisture.

The unweighted UniFrac PCoA (Fig. 1f and g) and PERMANOVA analyses (Table S1) showed that soil moisture and CFF application were the main drivers of soil β-diversity. The separation factor for the first principal coordinates for bacteria (8.81) and fungi (12.54) was CFF application, and the separation factor for the second principal coordinates for bacteria (5.36) and fungi (8.98) was soil moisture. Compared with the control group (PERMANOVA, bacteria: *P* = 0.03, *R*^2^ = 0.33; fungi: *P* = 0.27, *R*^2^ = 0.28), CFF application resulted in more significant differences in microbial community structure between the soil-moisture levels (PERMANOVA, bacteria: *P* = 0.001, *R*^2^ = 0.29; fungi: *P* = 0.001, *R*^2^ = 0.41); however, for each soil-moisture condition, the application rate of CFF had no significant impact on soil bacterial and fungal community composition. Therefore, in the following sections, the results obtained for different CFF application rates are the mean for each soil-moisture condition and considered as one treatment.

### Soil microbial community structure

**Fig. 2:**
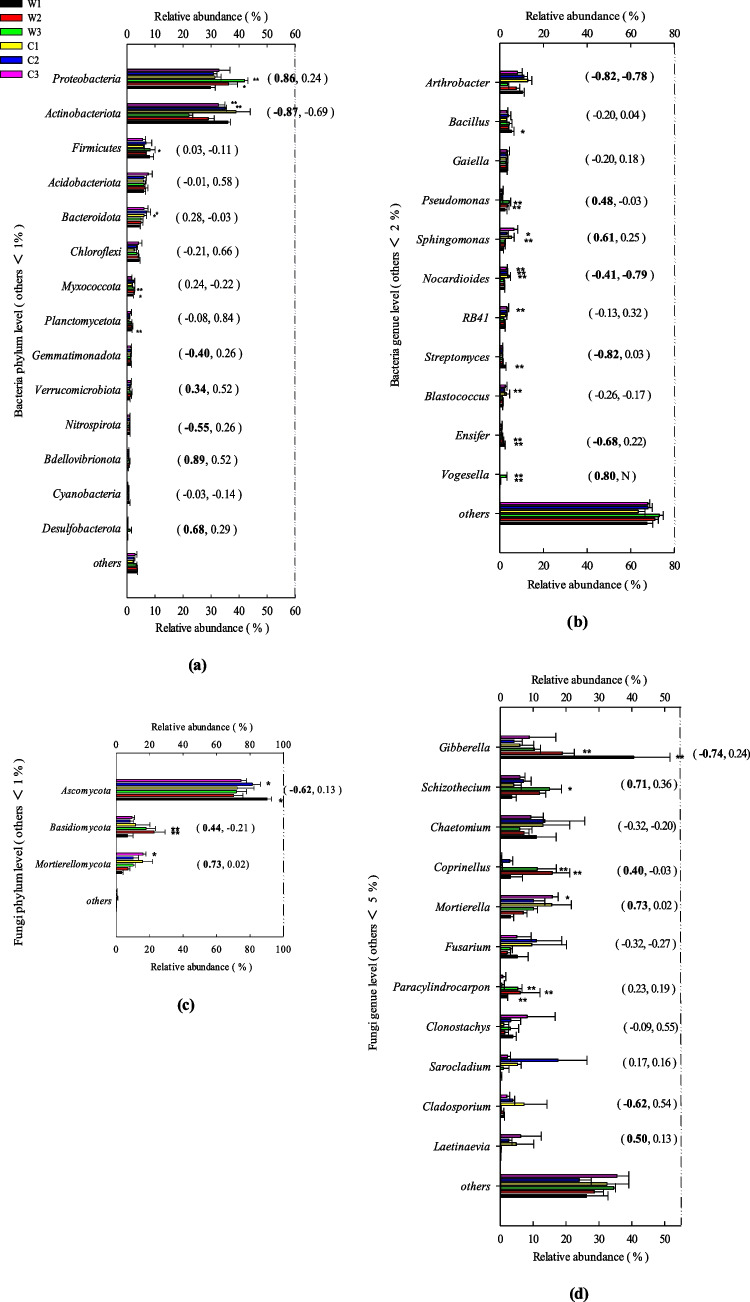
Structural changes at the phylum and genus levels of indigenous bacteria and fungi. The numbers in parentheses represent the correlation coefficients between relative abundance at the **a** bacterial phylum (others < 1%), **b** bacterial genus (others < 2%), **c** fungal phylum (others < 1%), and **d** fungal genus levels (others < 5%) and soil moisture for CFF application (left) and control treatments (right). Bold font indicates statistical significance (*P* < 0.05), where * and ** indicate significance at the 1% and 5% levels, respectively; N indicates the absence of a species in the corresponding treatment

A total of 45 bacterial phyla and 1126 bacterial genera were identified in the soil samples. This includes 14 abundant phyla (Fig. 2a), 28 abundant genera, and 11 other genera with relative abundances > 2% (Fig. 2b). At the phylum level, *Proteobacteria* (29.76–42.06%) and *Actinobacteriota* (32.58–38.86%) were dominant in the CFF-treated (W) and control (C) soils, respectively. Other dominant phyla included *Firmicutes* (W: 6.75–8.21%, C: 5.53–6.80%), *Acidobacteriota* (W: 5.98–6.42%, C: 5.93–7.59%), and *Bacteroidota* (W: 4.36–5.04%, C: 6.04–6.91%). These dominant bacterial phyla accounted for more than 80% of the sequences in the treatment and control groups (W: 83.33–84.14%, C: 84.49–88.11%). Regardless of CFF application, *Actinobacteriota* was significantly and inversely correlated with soil moisture (W: *r* =  − 0.87, *P* < 0.01; C: *r* =  − 0.69, *P* = 0.04). In the 20% and 30% moisture treatments with CFF application, the relative abundance of *Actinobacteriota* was significantly lower than that of the control group (*P* < 0.01), while the relative abundance of *Proteobacteria* was significantly higher (20%: W: 36.13%, *P* = 0.036; 30%: W: 42.06%, *P* = 0.004). Overall, *Proteobacteria* was significantly correlated with soil moisture (W: *r* = 0.81, *P* < 0.001). In the CFF-treated soils, the five bacterial genera with the highest relative abundances were *Arthrobacter* (3.45–10.22%), *Bacillus* (4.02–5.23%), *Pseudomonas* (2.29–4.33%), *Gaiella* (2.79–3.07%), and *Sphingomonas* (1.60–2.30%), accounting for more than 20% of the sequences. *Arthrobacter* and *Nocardioides* were significantly negatively correlated with soil moisture in both the treatment and control groups; *Pseudomonas* (*r* = 0.48, *P* = 0.003) and *Sphingomonas* (*r* = 0.61, *P* < 0.001) were significantly positively correlated with soil moisture in the CFF treatment group; and the relative abundance of *Pseudomonas* in the 20% (3.44%, *P* < 0.001) and 30% (4.33%, *P* = 0.002) moisture treatments was also significantly increased with CFF treatment.

A total of 10 fungal phyla and 452 fungal genera were detected, including three abundant phyla (Fig. 2c), 41 abundant genera, and 11 other genera with relative abundances > 5% (Fig. 2d). At the phylum level, *Ascomycota* had the highest relative abundance in both the CFF-treated (70.25–90.10%) and control (72.33–81.33%) groups, followed by *Basidiomycota* (W: 6.65–22.61%; C: 8.26–11.56%), which together accounted for more than 85% of the total sequences. The relative abundance of *Basidiomycota* was significantly increased with CFF application under the 20% and 30% moisture conditions. At genus level and compared with the control group, CFF application significantly increased the relative abundance of *Paracylindrocarpon*, *Pyrenochaetopsis*, and *Fusicolla* under all soil-moisture conditions but decreased the relative abundance of *Sarocladium*.

The addition of CFF also led to an increase in the number of correlations between different bacterial and fungal phyla/genera and soil moisture. For example, when treated with CFF, at the bacterial phylum level, *Verrucomicrobiota* (*r* = 0.34, *P* = 0.041), *Bdellovibrionota* (*r* = 0.89, *P* < 0.001), *Desulfobacterota* (*r* = 0.68, *P* < 0.001), *Gemmatimonadota* (*r* =  − 0.40,* P* = 0.017), and *Nitrospirota* (*r* =  − 0.55, *P* = 0.001) were all significantly correlated with soil moisture, and at the fungal phylum level, *Ascomycota* (*r* =  − 0.62, *P* < 0.01), *Basidiomycota* (*r* = 0.44, *P* < 0.01), and *Mortierellomycota* (*r* = 0.73, *P* < 0.01) were significantly correlated with soil moisture. Similar patterns were found at the genus level.

### Microbial network analysis

**Fig. 3:**
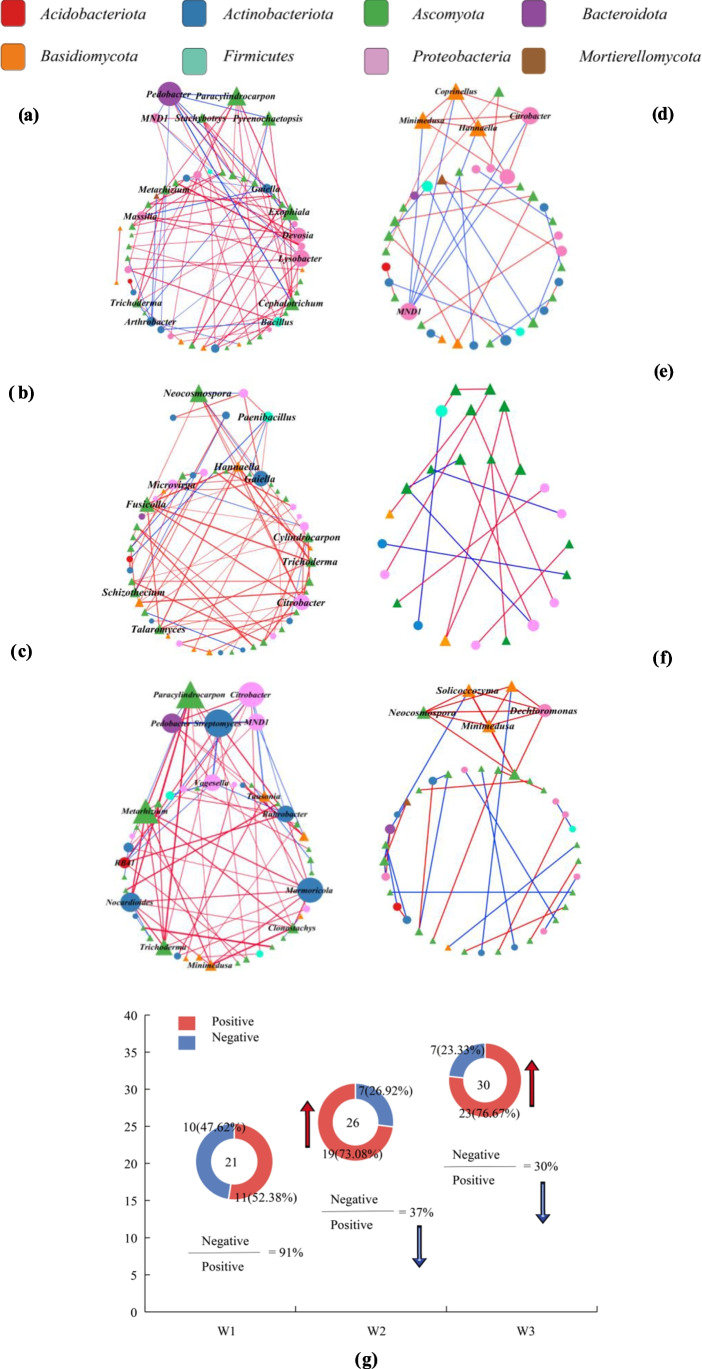
Network diagram of correlated bacterial and fungal genera for treatments **a** W1, **b** W2, **c** W3, **d** C1, **e** C2, and **f** C3. Treatment abbreviations are defined in Table 1. Nodes represent the genera involved in the network, with circles denoting bacterial genera and triangles denoting fungal genera. Node size indicates the degree of the node, and node color indicates phyla. Connecting edges represent significant correlations between genera, with red indicating a positive correlation (Spearman’s *r* > 0.7, *P* < 0.01) and blue indicating a negative correlation (Spearman’s *r* <  − 0.7,* P* < 0.01). Edge thickness represents the strength of the correlation. The five nodes marked above the circles in each graph are the top five genera for centrality for each treatment, and the nodes marked with the genera name are those with a node degree > 5 for each treatment. **g** The number of positive and negative correlations between indigenous bacteria and fungi. The numbers in the circles represent the number of correlations; the numbers above the circles represent the number and percentage of positive and negative correlations in the relationship; the ratios below the circles represent the ratios of percentages of positive and negative correlations

The application of CFF under all soil-moisture conditions increased the number of network nodes and edges in the soil microbial correlation network (Table S2). Specifically, the application of CFF increased the interactions between bacteria and fungi, both within and between species (nodes), and improved the connections (edges) between microbial taxa. The highest percentage of fungi-fungi with correlations was found in the CFF application for soil-moisture conditions of 10% (W1) and 20% (W2), with 44.32% and 41.33%, respectively (Supplementary Table 2). Compared to W1, the W2 treatment resulted in a stronger relationship between bacteria and bacteria (W1: 67.86%, W2: 88.89%), and between bacteria and fungi (W1: 52.38%, W2: 73.08%), and the relationships between fungi and fungi were always positive. Under the 30% soil-moisture treatment (W3), more bacteria-fungi correlations were observed, and a greater proportion of these was positive. Thus, the proportion of negative or positive correlations between bacteria and fungi increased with soil-moisture content (W1: 52.38%, W2: 73.08%, W3: 76.67%; Fig. 3g).

The taxa with a high degree of centrality and betweenness centrality are considered key taxa in microbial networks, playing potential leadership roles in specific network ecosystems. As shown in Fig. 3, applying CFF under different soil-moisture conditions changes the key taxa in the control treatment. Specifically, under the 10% moisture treatment (W1), *Pedobacter* of *Bacteroidota* had the highest centrality. The remaining genera with a degree of centrality > 5 mainly consisted of *Proteobacteria* and *Actinobacteriota* (Fig. 3a). Under the 20% moisture treatment (W2), *Neocosmospora* of *Ascomycota* had the highest centrality. The other key taxa were *Firmicutes*, *Proteobacteria*, and *Actinobacteriota* (Fig. 3b). Lastly, under the 30% soil-moisture treatment (W3), the key taxa were in *Ascomycota*, *Proteobacteria*, *Acidobacteriota*, *Actinobacteriota*, *Bacteroidota (RB41)*, and *Basidiomycota* (*Minimedusa*; Fig. 3c).

### Correlation analysis of bacteria and fungi related to corn straw degradation

**Fig. 4:**
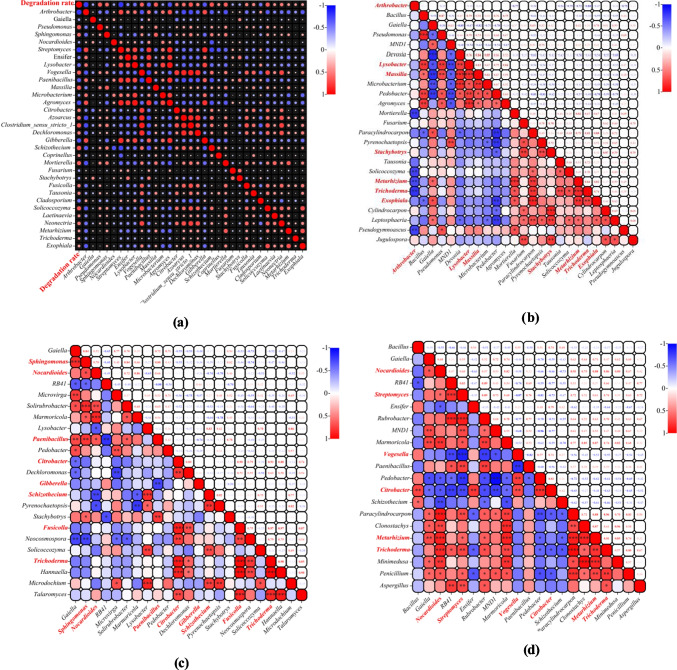
**a** Correlation analysis of bacteria and fungi related to corn straw degradation. Red and blue dots represent positive and negative correlations, respectively, and dot size represents the strength of the correlation (Spearman’s correlation coefficient). **b**,** c**, and **d** Correlation analysis of key genera related to corn straw degradation and their related genera for the CFF-treated soils with a moisture content of 10%, 20%, and 30%. Red and blue squares represent positive and negative correlations, respectively. The genera marked in red are the key genera influencing corn straw degradation rate. The numbers in the upper right are the correlation coefficients between genera (Spearman’s correlation coefficient), and the lower right indicates the significance of each correlation, where *P < 0.05; **P < 0.01; and ***P < 0.001

A total of 32 genera were significantly correlated with corn straw degradation rate (*P* < 0.05; Fig. 4a). Specifically, nine bacterial genera were significantly positively correlated with degradation rate, including *Pseudomonas* and *Sphingomonas*; eight bacterial genera were significantly negatively correlated with degradation rate, including *Arthrobacter* and *Gaiella*; 11 fungal genera were significantly positively correlated with degradation rate, including *Schizothecium* and *Coprinelluss*; and four fungal genera were significantly negatively correlated with degradation rate, including *Gibberella* and *Fusarium* (Table S3).

To further explore whether the changes in soil microbial community structure were related to the degradation of corn straw, we screened the key genera (with the top five centrality rankings and degree > 5) of the constructed networks (Fig. 3a–c) that correlated with corn straw degradation rate and revealed the interactions between these and related genera (Fig. 4b–d).

Figure 4b shows the interactions between the key genera and between correlated non-key genera under the W1 treatment. Most correlations were found between bacteria and bacteria or between fungi and fungi, while fewer were found between bacteria and fungi; for the W2 treatment (Fig. 4c), more correlations between the key bacteria and fungi were found relative to the W1 treatment; and for the W3 treatment (Fig. 4d), the number of associations between bacteria and fungi (15) was similar to that of the W2 treatment (17). Except for *Nocardioides* and *Schizothecium*, which were negatively correlated (*r* =  − 0.735, *P* = 0.007), all correlations were positive. Furthermore, compared with the W2 treatment, the number of non-key bacteria and fungi showing associations with the key bacterial genera increased substantially. Overall, consistent with the characteristics of the association network of indigenous microorganisms (Fig. 3), the level the interactions between bacteria and fungi related to corn straw degradation increased with soil moisture, and most of these interactions were positive.

## Discussion

### Effect of different treatments on the α-diversity of indigenous microorganisms

Microorganisms are extremely sensitive to environmental conditions, such as moisture, which controls their demographic parameters and activity (Aupic-Samain et al. 2021). Wu et al. (2022) found that the decrease in soil moisture caused by climate warming was a major driver of the decline in microbial diversity via changes in microbial activity and interactions. Xia et al. (2020) analyzed the effects of soil moisture, texture, and physicochemical properties on bacterial and fungal diversity and their composition, reporting that soil texture was more closely associated with fungal diversity than soil moisture. Evans and Wallenstein (2012) also conducted a series of soil drying and rewetting experiments and found that bacteria were more sensitive to changes in moisture than fungi owing to their dependence on water-film flow for movement and obtaining nutrients.

In our study, consistent with previous studies, the richness and diversity of indigenous bacteria in slightly wet (20%) and wet (30%) soil were higher than those in dry soil (10%) (Fig. 1b and c). To investigate the effects of the bacterial agent (CFF) and soil-moisture conditions on the observed differences in corn straw degradation rates, soil samples were collected from the same location and mixed evenly. Furthermore, we ensured that the moisture treatments were not extreme and that they were calibrated based on field conditions. Therefore, CFF application significantly increased the richness and diversity of bacteria but did not alter the fungal taxa in our study. Based on cost considerations, the CFF application rate was limited to 3 kg/667 m^2^ (800 kg straw), likely explaining the absence of significant differences in soil diversity and community structure between the different CFF application rates under the same soil-moisture conditions.

### Structural changes in indigenous bacterial and fungal communities

Moisture is an important factor in soil microbial community structure regulation, and microorganisms with different abilities to acquire water show different responses to water availability (Liu et al. 2021). In our study, the application of CFF strengthening the relationships between indigenous microorganisms and soil moisture altered the relative abundances of bacterial and fungal taxa at the phylum and genus levels (Fig. 2).

Barnard et al. (2013) sequenced the phylogenetic marker genes for potentially active soil bacterial and fungal communities in three annual grasslands in California, USA, and found that the abundance of *Ascomycota* increased under drought conditions and *Basidiomycota* existed in humid soils. Consistent with these findings, we found that in the CFF-treated soils, the abundance of *Ascomycota* was significantly inversely correlated with soil moisture, while the abundance of *Basidiomycota* was significantly positively correlated with soil moisture.

The change in dominant bacterial phyla after adding CFF can be explained by the oligotrophic-copiotrophic theory (Koch 2001). For instance, under nutrient-rich conditions, copiotrophs grow quickly and are dependent on a fertile environment, while oligotrophs grow slowly and prefer environments with low nutrient levels (Cheng et al. 2022). In addition, the degradation of corn straw, which is enhanced by an increase in soil moisture, releases nutrients (Latifmanesh et al. 2020). Therefore, as a copiotrophic bacterial phylum, *Proteobacteria* was enriched in the CFF-treated soils and was positively correlated with soil moisture. In contrast, *Actinobacteriota*, an oligotrophic phylum (Wang et al. 2022), had a lower relative abundance than the control treatment, showing a significant negative correlation with soil moisture. The oligotrophic bacteria *Nitrospirota* and *Gemmatimonadetes* (Roller and Schmidt 2015; Wan et al. 2020) also exhibited similar characteristics. These results show that applying bacterial agents, especially in soils with high moisture content (i.e., 20–30%), could increase the relative abundance and activity of copiotrophic bacterial taxa (Zhang et al. 2021).

Although the application of CFF significantly altered the relative abundance of indigenous bacterial and fungal communities in this study, the indigenous microorganisms were highly stable (Ratzke et al. 2020). Hence, the exogenous bacterial agent CFF possibly had an indirect effect, while the soil ecology altered by the accelerated degradation of corn straw by CFF was the direct factor that changed the structure of the indigenous microbial community. Said-Pullicino et al. (2014) showed that accelerated degradation of straw significantly improved soil nitrogen (N) mineralization and N use efficiency, and organic acids released during straw decomposition could activate inorganic phosphorus, thus increasing the effectiveness of soil nutrients; Turmel et al. (2014) made a similar argument that straw re-degradation is an important way to improve soil fertility, increase soil organic carbon, and release nitrogen and other nutrients back into the soil. Soil microorganisms are very sensitive to the environment and can respond quickly to changes in the environment; therefore, they are selected as early biological indicators of soil quality (Chen et al. 2017). Yang et al. (2016) showed that SOC, AK, and TN are important factors affecting the structure of soil microbial communities. Su et al. (2020) pointed out that straw return could provide soil microbial communities with appropriate nutrients and salinity to improve their abundance and structure.

Microorganisms grown in different environments are actively selective; i.e., different microbial species are suitable for growth in different environmental conditions, or the relative abundance of the same microbial species in different environments varies (Kandasamy et al. 2019). In this study, application of bacterial agents to different soil-moisture conditions accelerated the degradation process of corn straw. Different levels of straw degradation with different nutrient release capacities and metabolites created different soil biological environments. The nutrients and metabolites released by straw degradation drive the corresponding soil microbial enrichment, which in turn changes the abundance of microflora. The increase in abundance and enzyme activity is also the main factors driving straw degradation. Empirical applications have shown that external factors can shape the microbiome with different functional structures and that microbiomes with different functional structures can also influence soil function (Ellouze et al. 2013). This also provides an idea for the research on the use of bacterial agents to modulate soil microorganisms to improve straw degradation. Studying whether the application of exogenous bacterial agents can promote straw degradation by changing the structure of indigenous flora, specifically those related to straw degradation function, is a new idea to realize the effective application of exogenous bacterial agents and an important aspect to reveal the degradation mechanism of corn straw degrading from the perspective of soil microorganisms.

### Microbial networks

The coexistence of bacteria and fungi in soil forms a complex system of species interactions (Mangse et al. 2020). Microbial network analysis can effectively reveal the coexistence of relationships and key species in complex microbial systems (Wang et al. 2021). In our study, when treated with CFF, the key soil bacterial taxa were *Actinobacteriota*, *Bacteroidota*, and *Proteobacteria*, and the key fungal taxa were *Ascomycota* and *Basidiomycota* (Fig. 3). Using ^13^C-labeled rice straw to examine microbial degradation, Shrestha et al. (2011) found that *Proteobacteria*, *Actinobacteriota*, and *Bacteroidota* were dominant. *Actinobacteriota* is known for utilizing lignin-derived compounds to generate cellular-degrading enzymes that further enhance lignin degradation (Kirby 2005). Zhao et al. (2020) also analyzed the microorganisms involved in the decomposition of soil leaf litter from three macrophyte species. They found that the fungi *Ascomycota* and *Basidiomycota* were the dominant phyla, accounting for 60–70% of all sequences, and *Basidiomycota* played a dominant role in the degradation of lignin components. In addition, Ma et al. (2013) found that *Ascomycota* played a leading role at different stages of straw degradation. These phyla are all involved in the decomposition of various types of lignocellulose, indicating that they are the key taxa driving straw degradation.

Yi et al. (2022) investigated the interactions between soil microbial communities under benzo[a]pyrene (BaP) stress. They showed that to reduce BaP pollution stress, the abundance of aromatic compound-degrading microbes increased alongside enhanced intraspecific bacterial community cooperation. In our study, the addition of CFF significantly increased the degradation rate of corn straw (Fig. 1a). More associations within and among bacterial and fungal genera related to straw degradation were enriched in the indigenous microbial association network (Fig. 3). Furthermore, with an increase in soil moisture, the proportions of positive intraspecific and interspecific correlations significantly increased that positive bacterial-fungal associations increased from 52.38% in the 10% moisture treatment to 73.08% and 76.67% in the 20% and 30% soil-moisture conditions, respectively. Similar patterns were found in the correlations between the key straw-degrading taxa (Fig. 4). Based on these results, we can infer that adding CFF improves corn straw degradation rates by enriching corn straw-degrading bacteria and fungi and promoting positive interactions in the ecological network. Coupled with increasing soil moisture, this approach offers a valuable strategy for improving the degradation rate of corn straw in low-temperature soils.

In summary, some microorganisms that can better adapt to their environment may survive and become dominant, substantially changing the coexistence patterns of the species (Naylor and Coleman-Derr 2018) and, consequently, the structure and diversity of microbial communities. Changes are unified and coordinated processes. Therefore, the mechanism by which bacterial agents, such as CFF, alter corn straw degradation rates under different soil-moisture conditions is strongly associated with changes in the interactions between multiple microbial species rather than the effect of a single species or factor.

## Supplementary Information

Below is the link to the electronic supplementary material.

Supplementary file1 (PDF 157 KB)

## Data Availability

All data generated or analyzed during this study are included in this published article (and its supplementary information files). The datasets generated during and/or analyzed during the current study are available from the corresponding author on reasonable request.
