# Supplementary material for: Low-temperature corn straw-degrading bacterial agent and moisture effects on indigenous microbes
Source: Appl Microbiol Biotechnol. 2023 Jul 1;107(16):5241–55. doi: 10.1007/s00253-023-12644-8 (PMC10386949; doi:10.1007/s00253-023-12644-8)
Supplement: Supplementary file 1 — Supplementary file1 (PDF 157 KB) [file 253_2023_12644_MOESM1_ESM.pdf]

Applied Microbiology and Biotechnology

Low-temperature corn straw-degrading bacterial agent and moisture effects on indigenous microbes

Sainan Zhang[1], Shengcai Han[2,3], Julin Gao[1,2*], Xiaofang Yu[1,2*], Shuping Hu[1,2]

[1]*College of Agriculture, Inner Mongolia Agricultural University, Hohhot 010000, People's Republic of China*

[2]*Key Laboratory of Crop Cultivation and Genetic Improvement, Inner Mongolia Autonomous Region, Hohhot 010000, People's Republic of China*

[3]*College of Horticulture and Plant Protection, Inner Mongolia Agricultural University, Hohhot 010000, People's Republic of China*

---

* **Corresponding Author:** Julin Gao, 306 Zhaowunda Road, Saihan District, Hohhot City, Inner Mongolia Autonomous Region, PR China. Phone: +86-13704753317, Fax: (0471) 430-1530, E-mail: gjl1103268342@163.com; Xiaofang Yu, 306 Zhaowunda Road, Saihan District, Hohhot City, Inner Mongolia Autonomous Region, PR China. Phone: +86-13294815858, Fax: (0471) 431-8142, E-mail: yxf1103268342@163.com.

Supplementary Information

Table 1. Statistical significance of differences (PERMANOVA)

Group			Sample	*P*	R2
Bacteria	W1		1, 2, 3, 4	0.91	0.22
	W2		5, 6, 7, 8	0.28	0.3
	W3		9, 10, 11, 12	0.32	0.28
	WC	W	1, 2, 3, 4, 5, 6, 7, 8, 9, 10, 11, 12	0.001	0.18
		C	C1, C2, C3		
	W	W1	1, 2, 3, 4	0.001	0.29
		W2	5, 6, 7, 8		
		W3	9, 10, 11, 12		
	C	C1	C1	0.033	0.33
		C2	C2		
		C3	C3		
Fungi	W1		1, 2, 3, 4	0.17	0.33
	W2		5, 6, 7, 8	0.48	0.27
	W3		9, 10, 11, 12	0.05	0.38
	WC	W	1, 2, 3, 4, 5, 6, 7, 8, 9, 10, 11, 12	0.001	0.24
		C	C1, C2, C3		
	W	W1	1, 2, 3, 4	0.001	0.41
		W2	5, 6, 7, 8		
		W3	9, 10, 11, 12		
	C	C1	C1	0.27	0.28
		C2	C2		
		C3	C3		

W1, W2, W3: Test for statistical significance of differences between the application rate of the bacterial agent at 10%, 20%, and 30% soil moisture. WC: Test for the statistical significance of differences between treatment and no-treatment. W: Test for the statistical significance of differences in soil moisture upon application of the bacterial agent (CFF). C: Test for the statistical significance of differences in soil moisture without the application of the bacterial agent. *P*: Significant difference value, $P < 0.05$ indicates a statistically significant difference.

Table 2. Properties of the nodes and edges of the network graphs

T	A	B	C	D	E (%)	F	G (%)	H	I (%)
			B-B	28	31.82	19	67.86	9	32.14
W1	54	88	F-F	39	44.32	36	92.31	3	7.69
			B-F	21	23.86	11	52.38	10	47.62
			B-B	18	24.00	16	88.89	2	11.11
W2	56	75	F-F	31	41.33	31	100.00	0	0.00
			B-F	26	34.67	19	73.08	7	26.92
			B-B	26	34.21	16	61.54	10	38.46
W3	46	76	F-F	20	26.32	17	85.00	3	15.00
			B-F	30	39.47	23	76.67	7	23.33
			B-B	6	16.67	2	33.33	4	66.67
C1	39	36	F-F	10	27.78	8	80.00	2	20.00
			B-F	20	55.56	11	55.00	9	45.00
			B-B	1	5.88	0	0.00	1	100.00
C2	24	17	F-F	7	41.18	6	85.71		14.29
			B-F	9	52.94	6	66.67	3	33.33
			B-B	4	10.53	2	50.00	2	50.00
C3	41	38	F-F	17	44.74	14	82.35	3	17.65
			B-F	17	44.74	7	41.18	10	58.82

T: Different treatments. A: Number of nodes. B: Number of edges. C: Category of species with correlation. B-B: Bacteria–Bacteria. F-F: Fungi–Fungi. B-F: Bacteria–Fungi. D: Number of species with correlations. E: Percentage of species with correlations. F: Number of species with positive correlation. G: Percentage of species with positive correlations. H: Number of species with negative correlations. I: Percentage of genera with negative correlations.

Table 3. Genera significantly associated with the degradation rate of corn straw

Bacteria	r	*P*	Fungi	r	*P*
Arthrobacter	-0.780	0.000	*Gibberella*	-0.659	0.000
Gaiella	-0.359	0.031	*Schizothecium*	0.678	0.000
Pseudomonas	0.656	0.000	*Coprinellus*	0.433	0.008
Sphingomonas	0.579	0.000	*Mortierella*	0.637	0.000
Nocardioides	-0.358	0.032	*Fusarium*	-0.353	0.035
Streptomyces	-0.795	0.000	*Stachybotrys*	-0.355	0.034
Ensifer	-0.464	0.004	*Fusicolla*	0.510	0.001
Lysobacter	-0.562	0.000	*Tausonia*	0.482	0.003
Vogesella	0.633	0.000	*Cladosporium*	-0.546	0.001
Paenibacillus	-0.619	0.000	*Solicoccozyma*	0.645	0.000
Massilia	0.650	0.000	*Laetinaevia*	0.434	0.008
Microbacterium	-0.391	0.018	*Neonectria*	0.533	0.001
Agromyces	-0.688	0.000	*Metarhizium*	0.406	0.014
Citrobacter	0.519	0.001	*Trichoderma*	0.375	0.024
Azoarcus	0.572	0.000	*Exophiala*	0.404	0.015
Clostridium_sensu_stricto_1	0.679	0.000			
Dechloromonas	0.513	0.001			

r: Correlation coefficients (Spearman) of genus and corn straw degradation rates. Positive numbers indicate a positive correlation; negative numbers indicate a negative correlation. *P*: Significant difference value, $P < 0.05$ indicates a statistically significant difference.
